# *Plasmodium vivax* Antigen Discovery Based on Alpha-Helical Coiled Coil Protein Motif

**DOI:** 10.1371/journal.pone.0100440

**Published:** 2014-06-24

**Authors:** Nora Céspedes, Catherine Habel, Mary Lopez-Perez, Angélica Castellanos, Andrey V. Kajava, Catherine Servis, Ingrid Felger, Remy Moret, Myriam Arévalo-Herrera, Giampietro Corradin, Sócrates Herrera

**Affiliations:** 1 Malaria Vaccine and Drug Development Center (MVDC), Cali, Colombia; 2 School of Health, University of Valle, Cali, Colombia; 3 Biochemistry Department, University of Lausanne, Epalinges, Switzerland; 4 Caucaseco Scientific Research Center, Cali, Colombia; 5 Fundación Centro de Primates, Cali, Colombia; 6 Centre de Recherches de Biochimie Macromoleculaire (CRBM) and Institut de Biologie Computationnelle (IBC), CNRS, University of Montpellier, Montpellier, France; 7 University ITMO, St. Petersburg, Russia; 8 Swiss Tropical and Public Health Institute, Basel, Switzerland; 9 Hôpital Saint Camille, Ouagadougou, Burkina Faso; Ehime University, Japan

## Abstract

Protein α-helical coiled coil structures that elicit antibody responses, which block critical functions of medically important microorganisms, represent a means for vaccine development. By using bioinformatics algorithms, a total of 50 antigens with α-helical coiled coil motifs orthologous to *Plasmodium falciparum* were identified in the *P. vivax* genome. The peptides identified *in silico* were chemically synthesized; circular dichroism studies indicated partial or high α-helical content. Antigenicity was evaluated using human sera samples from malaria-endemic areas of Colombia and Papua New Guinea. Eight of these fragments were selected and used to assess immunogenicity in BALB/c mice. ELISA assays indicated strong reactivity of serum samples from individuals residing in malaria-endemic regions and sera of immunized mice, with the α-helical coiled coil structures. In addition, *ex vivo* production of IFN-γ by murine mononuclear cells confirmed the immunogenicity of these structures and the presence of T-cell epitopes in the peptide sequences. Moreover, sera of mice immunized with four of the eight antigens recognized native proteins on blood-stage *P. vivax* parasites, and antigenic cross-reactivity with three of the peptides was observed when reacted with both the *P. falciparum* orthologous fragments and whole parasites. Results here point to the α-helical coiled coil peptides as possible *P. vivax* malaria vaccine candidates as were observed for *P. falciparum*. Fragments selected here warrant further study in humans and non-human primate models to assess their protective efficacy as single components or assembled as hybrid linear epitopes.

## Introduction

Despite the important reduction in reported malaria incidence during the last decade in a number of countries worldwide, malaria infection still represents one of the major global public health threats. The World Health Organization (WHO) estimated an annual global burden of 207 million malaria cases and 627,000 deaths in 2012 [1].

Of at least six different malaria parasite species which can be transmitted to humans, *Plasmodium vivax* is the second most parasite species of epidemiological importance with 70–80 million cases estimated per year worldwide [2]. In most malaria-endemic areas, it coexists with *P. falciparum*, thus making its control more difficult.

Due to the limited impact and cyclical loss of effectiveness of some of the classical malaria control measures, and based on multiple evidence on the feasibility of malaria vaccines, significant efforts have been invested in the development of malaria subunit vaccines over the past 2 to 3 decades [3]–[5]. Significant progress has been achieved with *P. falciparum* where several vaccine candidates are currently in clinical development [6]; with one now being considered for licensure [7]. In contrast, development of *P. vivax* vaccines has been significantly neglected and only a few candidates have been selected for clinical testing [8].

Most *P. vivax* antigens considered to have vaccine potential have been tested in *in vitro* studies as well as in preliminary preclinical studies in mice and primates [9]–[13]. Only a few of these antigens further selected by classical immuno-serological methods have undergone phase I clinical trials [14]–[16]. In the past, the number of parasite antigens available for vaccine studies has been quite limited. Presently, advances in the establishment of *Plasmodium* genomes and proteomes [17]–[19] together with high throughout laboratory techniques [20], can potentially accelerate the development of malaria vaccines. Additionally, the use of bioinformatics tools to explore the malaria genome/proteome databases has allowed new approaches for identification of parasite proteins containing α-helical coiled coil domains [21].

Such domains readily fold into stable structures that are capable of eliciting antibodies reactive with structurally native epitopes, and are generally monomorphic [22]; these structures have the capacity to block critical functions of medically important microorganisms [23], [24]. Specifically in *P falciparum* some antigens containing these domains have been involved in antibody-dependent inhibition of malaria parasite growth [25], [26], and therefore represent targets for vaccine development, thus drastically reducing the time required for antigen selection and preclinical testing [21].

In the past few years, approximately 170 *P. falciparum* α-helical coiled coil protein fragments have been assessed by combining genome-wide bioinformatics analysis, peptide selection, peptide chemical synthesis, immune and biochemical assays, *in vitro* functional assays, with associated protection analysis [25], [26] (unpublished data). A total of 140 putative α-helical coil-containing proteins of 200 to 10,000 amino acids in length were identified as new target proteins in *P. falciparum* asexual blood stages. Here we describe studies carried out using the same technology and approach with *P. vivax* antigens orthologous to *P. falciparum*, which have been evaluated for their antigenicity using human sera and immunogenicity in mice.

## Materials and Methods

### *P. vivax* genome bioinformatics analysis

Orthologues are good candidates for multi-species vaccines as they have the potential to elicit antigenic reactions against all the species included in the search parameters. A *P. vivax* Salvador I genome database (PlasmoDB) was used for the selection of *P. vivax* orthologous to *P. falciparum* protein sequences from asexual blood stages containing α-helical coiled coil structures, analyzed by COILS software [27]. Fifty *P. vivax* orthologues were found to have at least 30% homology with the 170 *P. falciparum* α-helical coiled-coil proteins previously identified. Sequences were of the maximal length possible in order to maximize the stability of the α-helical conformations and to increase the array of conformational epitopes that could be yielded. Selected α-helical coiled coil-containing proteins were further characterized as to possible surface location and GPI anchoring, using the following software: identification of potential signal peptides by SecretomeP and SignalP (http://www.cbs.dtu.dk/services/) [28]; transmembrane spanning region- (TMPRED http://www.ch.embnet.org/ software/TMPRED_rm.html and TMHMM http://www.cbs.dtu.dk/services/TMHMM; [29], and GPI-anchored proteins (http://mendel.imp.univie.ac.at/sat/gpi/gpi_server.html [30]; and prediction of sub-cellular localization (pTARGET) [31]. Additionally, major histocompatibility complex protein (MHC-II) binding predictions were made using the IEDB analysis resource Consensus tool [32], [33] which combines predictions from ANN aka NetMHC [34], [35], SMM [36] and Comblib [37] within the sequence of preselected peptides used in murine immunogenicity studies.

### Peptide synthesis

Fifty *P. vivax* polypeptides 25 to 57 amino acids long were synthesized by fluorenylmethoxycarbonyl (F-moc) solid-phase chemistry [38] using an Intavis AG Bioanalytical synthesizer (Germany) (Table S1). The resulting construct was HPLC-purified; purity was confirmed by analytic C18 HPLC and mass spectrometry (MALDI-TOF; Applied Biosystem, Foster City, CA). All reagents were purchased from Fluka (Buchs, Switzerland) and Novabiochem (Laufelfingen, Switzerland). Additionally, five *P. falciparum* polypeptides (*Pf-*P27, *Pf-*P43, *Pf-*P45, *Pf-*P82 and *Pf-*P96) described previously [26] were used to test cross-reactivity between *P. vivax* and *P. falciparum* species.

### Circular dichroism studies

Spectra of peptides were recorded on a JASCO J-810 spectrometer (JASCO corporation, Tokyo, Japan) equipped with a temperature controller and a 0.1 cm path length cuvette. The measurements were made in water at pH 7.3 and 22°C and at a peptide concentration of 0.15 mg/mL.

### Human sera

Human serum samples from adults living in malaria-endemic areas of Colombia and Papua New Guinea (PNG) as well as from a non-endemic area (Switzerland) were used to assess peptide antigenicity. Sera (n = 42) were collected from Maprik District of the East Sepik Province, a malaria-endemic region of PNG, during a cross-sectional survey described previously [39], whereas the Colombian samples (n = 90) were obtained from two geographically distant and epidemiologically different malaria-endemic sites: Tumaco (Nariño state, n = 51) and Tierralta (Córdoba state, n = 39). Previous infection with *P. vivax* was confirmed based on a positive *P. vivax* blood-stage immunofluorescent antibody test (IFAT) result. Ethical clearances for this study were obtained from the PNG Medical Research Advisory Committee as well as from the Institutional Review Boards (IRB) of the Malaria Vaccine and Drug Development Center–MVDC (CECIV) in Cali, Colombia. Written informed consent (IC) was obtained from each volunteer. Negative control samples were obtained from Swiss adult donors with no history of malaria and no previous travel to malaria-endemic areas. Human antibodies specific to *Pf-*P27 and *Pf*-P45 [26], were affinity-purified from a pool of human serum samples from adults living in Burkina Faso, and used to test cross-reactivity to the respective *P. vivax* orthologues.

### Animals and immunization procedures

Five-week old female BALB/c mice, maintained at the facility of MVDC and handled according to institutional guidelines, were divided into eight groups of four animals each. Mice were injected three times with the selected antigens formulated in Montanide ISA 720 adjuvant (Seppic Inc., Paris, France). Each mouse was injected subcutaneously at the base of the tail with 20 µg of the peptide formulation in a final volume of 50 µL on days 0, 20 and 40. Approximately 150 µL of whole blood were collected eight days before the first immunization, and ten days after second and third immunizations, under anesthesia from the orbital sinus; antibody responses were measured by ELISA as described previously [40]. Twenty days after the final immunization, mice were euthanized by anesthetic inhalation and spleens and lymph nodes were aseptically removed. Mononuclear cells were obtained by lymph node and spleen maceration followed by separation using Ficoll-hystopaque gradients; cells were assayed immediately. IFN-γ production by mononuclear cells was determined using a specific ELIspot assay as described below.

### Ethics Statement

This study was carried out in strict accordance with institutional guidelines. The protocol was approved by the Committee on the Ethics of Animal Experiments of the Universidad del Valle (Permit Number: 004-08). All surgery was performed under anesthesia, and all efforts were made to minimize suffering.

### ELISA test

Antibody responses to the tested antigens were measured in human and murine sera by ELISA as described previously [40]. Briefly, ELISA plates (Nunc-Immuno Plate, Thermo, USA) were coated with 5 µg/mL of the respective polypeptides overnight. Plates were then blocked with 5% skim milk in PBS+0.05% tween-20 (PBST) pH 7.4 for 2 h at room temperature. After washing, plates were incubated 1 h at room temperature with sera samples prepared in PBST/2.5% skim-milk as follows: human sera were tested at a 1∶200 dilution, whereas murine sera were tested at three-fold serial dilutions starting at 1∶100. IgG antibodies were detected using alkaline phosphatase-conjugated anti-human or anti-mouse immunoglobulin (Sigma Chemical Co., St Louis, MO) at a 1∶1000 dilution. Enzymatic activity was developed after incubation for 30 min at room temperature with para-nitrophenyl phosphate substrate. The final reaction was read at 405 nm in a microplate reader (MRX, Dynex Technologies, Inc., Chantilly, VA). Cut-off points were calculated as three SD above the mean absorbance value of sera from healthy malaria- naïve Swiss volunteers or naïve mice, respectively. Positive responders were classified according to the OD ratio (OD values of tested sample divided by the cut-off value). Results were considered positive when absorbance of the test sera was higher than or equal to the cut-off points. All ELISA experiments were performed in duplicates in two independent experiments.

Since all fragments were orthologous to *P. falciparum*, we tested the cross-reactivity to this species using *P. falciparum* antigens and sera from mice immunized with *P. vivax* α-helical coiled coil fragments (*Pv*Pep27, *Pv*Pep43, *Pv*Pep45, *Pv*Pep82 and *Pv*Pep96). Likewise, we tested the *P. vivax* fragments with affinity-purified human IgG specific to *Pf-*P27 and *Pf-*P45, two *P. falciparum* fragments which had previously shown capacity to induce strong monocyte-dependent parasite killing [26]. As negative control, a different α-helical coiled coil non-related antigen was used.

### IFA test

Parasite recognition by anti-peptide antibodies was determined by IFAT, using as antigen, *P. vivax* blood stages obtained from Colombian patients, and mouse sera collected 10 days after last peptide immunization. Briefly, parasites were incubated with sera diluted 1∶20. This reaction was developed with fluorescein-conjugated goat anti-mouse IgG (Jackson Immunoresearch Laboratories, Inc., Baltimore, MD) diluted 1∶1000. Slides were mounted in 30% glycerol and examined under a Nikon Eclipse microscope by epifluorescence. *P. falciparum* parasite cross-reactivity was also determined by IFAT using as antigen, *Pf*-FCB-1 blood-stage parasites derived from *in vitro* cultures [41].

### Cellular immune responses in mice

To determine the potential of eight selected peptides to stimulate T-cell responses, *in vitro* IFN-γ-production by lymph nodes and splenocytes obtained from immunized mice was quantified. For this purpose a commercial mouse anti-IFN-γ ELISpot kit (Mabtech AB, Stockholm, Sweden) was used; the test carried out according to the manufacturer's instructions. Multiscreen 96-well plates (Millipore, Bedford, MA) were coated overnight at room temperature with 5 µg/mL anti-mouse IFN-γ antibodies. RPMI 1640 medium containing 10% fetal bovine serum (FBS, GIBCO) was used as a blocking solution. Freshly isolated mononuclear cells were plated into duplicate wells at 5×10^5^ cells in RPMI 1640 medium supplemented with 10% FBS (100 µL/well). Culture medium alone, Conconavalin A or 10 µg of each synthetic peptide/mL medium (100 µL/well) was added and plates were cultured for 40 h at 37°C in a 5% CO_2_ humidified atmosphere. After washing, biotinylated antibody at 1 µg/mL was added and incubated for 2 h at room temperature. Plates were washed and alkaline phosphatase-streptavidin (Mabtech AB, Stockholm, Sweden) was added (1∶1000). Spots were visualized by adding 50 µL/well of BCIP/NBT (Sigma), scanned and counted using the AID ELISpot reader (AID Autoimmun Diagnostika GmbH, Germany) to determine the number of spots/well. Results were expressed as the mean number of IFN-γ spot-forming cells (SFC) per 10^6^ cells.

### Statistical analysis

Fisher's exact test (2×2 contingency tables) was used to compare differences in seroprevalence between the PNG and Colombian groups; the ANOVA test was used to compare groups. Dunnett's Multiple Comparison Test was used as post-hoc analysis and p value<0.05 was considered statistically significant. Statistical analyses were performed using GraphPad Prism software (version 5.01; GraphPad Software Inc., San Diego, CA, USA.

## Results

### *P. vivax* genome bioinformatic analysis

A total of 50 *P. vivax* fragments, 25–57 residues long and containing the α-helical coiled coil motifs, were selected based on proteome and transcriptome data of *P. falciparum* orthologues present in erythrocytic parasite stages (Tables 1 and S1). Variable homology (29 to 100% identity) was observed between *P. falciparum* and the corresponding *P. vivax* fragments (Table S1), most of which (32 antigens) were greater than 60% homologous. Identification of potential signal peptides, transmembrane (TM) regions, and GPI-anchored or sub-cellular localization prediction revealed five proteins containing TM domains (*Pv*Pep39, *Pv*Pep101, *Pv*Pep122, *Pv*Pep123 and *Pv*Pep131) and another three involved in secretory pathways (*Pv*Pep52, *Pv*Pep60, *Pv*Pep96.01). These latter peptides also contained a signal peptide. One of the proteins is predicted to be located in the mitochondria (*Pv*Pep39); none contained a GPI anchor.

**Table 1:** Bioinformatics analysis of coiled coil fragments.

Peptide	MW	*P. vivax*	*P. falciparum*	aa sequence	Position	Cell localization/function	Coiled coil Domain
PvPep5	2936	PVX_003585	PFB0145c	IADIKISLEKLKYEVKDKKDCLENV	203–227	Hypothetical protein	84–380
PvPep12	5039	PVX_003585	PFB0145c	YKKELEEKAKIIEDLKDKICTLTNEVMDLKNVKNELAERDSSL	1023–1065	Hypothetical protein	1016–1244
PvPep27	3169	PVX_113335	MAL6P1.37	KKQNAEKELSVLKKNYDAMSEEIEEIT	654–680	Hypothetical protein	636–743
PvPep40	3634	PVX_119385	PFC0235w	NETIQRMSNSLLKYEQDIETYQNEVSTLTGK	675–705	Hypothetical protein	594–744
*Pv*Pep42	3348	PVX_087730	PF07_0014	NTPDYYKKITTKLQNNINNVEEYINNITNDINILKSSID	154–192	Hypothetical protein	164–259
*Pv*Pep43	4583	PVX_089660	PFD0685c	SVDINALNEQVKKLREELNKVTNEYDDFKNKLELLYQK	779–816	Chromosome associated protein	726–917
*Pv*Pep45	4333	PVX_123385	PF11_0207	KEVKVEVNEVGEEVNEVKEEVNEAKEEVIEKKEEMTE	650–686	Hypothetical protein	557–781
*Pv*Pep52	3617	PVX_123480	PFL0770w	VEQVKKEINQINEQININETKITHLRNKIE	176–205	Secretory pathway	166–207
*Pv*Pep63	3658	PVX_118160	PF07_0086	NNEMDETLSKLKKDINKLNEKIQKYDNYVK	207–236	Hypothetical protein	162–244
*Pv*Pep82.02	6721	PVX_122740	MAL13P1.96	ETINQIDQKMEEIENNINLALEELKNLDQKILELQASFTCYENEIKQVIKKIEGLEK	862–918	Structural maintenance of chromosome 2	980–1045
*Pv*Pep82.03	6574	PVX_091910	MAL13P1.96	IEQLNTKMKNINENSNDSEHVNLAEFELKIAELKEDVNNINNMMKTFEMKFSALEK	471–526	Kelch domain-containing protein	462–551
*Pv*Pep83	4536	PVX_087730	PFC0345w	LQNNINNVEEYINNITNDINILKSSIDDERNERIIYNN	166–203	Hypothetical protein	164–259
*Pv*Pep90	4164	PVX_00072	PFD0520c	TRRMHSELSDGNKELKKLKKNIVQSDVLNAQLELNI	63–98	Hypothetical protein	64–98
*Pv*Pep95	3512	PVX_117455	PF14_0574	EKGLKDLNDKIRNYDSIIENQKKELEHLK	145–173	Hypothetical protein	145–245
*Pv*Pep96.01	6595	PVX_124060	PF13_0107	VEAVPENAEAAPENADPVHENAEAAPENAEPVHENAE	773–809	Secretory pathway	773–809
*Pv*Pep96.03	4482	PVX_084385	PF13_0107	DVQRIDTINKNISTINDDVDHINSNINNINDNLHKINSH	2051–2089	Hypothetical protein	2049–2088
*Pv*Pep101	3554	PVX_085155	PF14_0255	NKLTEMRRKLKIIDEKVQSVYKAIHAVLNN	314–343	CorA-like Mg2+ transporter protein	313–343
*Pv*Pep106	3441	PVX_114430	MAL6P1.163	KTIDQLDFEINDLNSKLKNYEKSVSQNKK	673–701	Hypothetical protein	430–799
*Pv*Pep123	3455	PVX_117855	PF14_0500	EKYSLIKEEIKYLNEDLDDLDNSVNVVKK	43–71	Hypothetical protein	40–86
*Pv*Pep125	3092	PVX_099410	PFI0975c	ILRKIEHSLKGWEADYNELKGKYNSV	1990–2015	Hypothetical protein	1924–2082

### Circular dichroism studies

**Figure 1:**
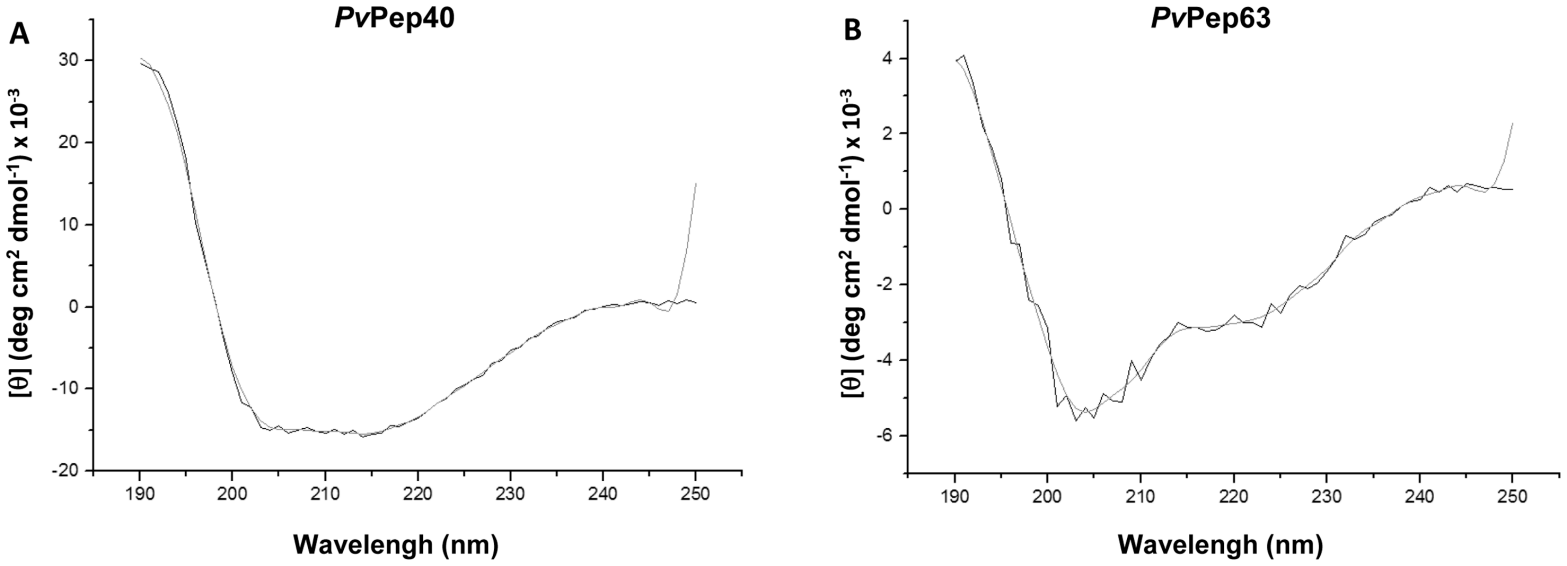
Representative CD spectra of peptides (A) *Pv*Pep40 and (B) *Pv*Pep63. The CD's were done at room temperature on 150 µg/mL samples in aqueous solutions. Spectrums came from the averages of duplicates in the far UVs, from 190 to 250 nm and were smoothed with a 5 points filter.

Circular dichroism (CD) studies of 16 randomly selected peptides indicate that they assume a total or partial α-helical conformation in water. Peptides 40-43, 55, 60 and 65 exhibit a CD pattern characteristic of a high α-helical content as indicate for *Pv*Pep40 (Figure 1A), whereas the remaining peptides (2, 5, 12, 27, 41, 45, 48 59 and 63) show CD profiles similar to that shown for peptide *Pv*Pep63 (Figure 1B) or intermediate between those shown in Figures 1A and 1B, all characteristic of a partial α-helical organization.

### Recognition of α-helical coiled coil peptides by human sera

Out of the 50 α-helical coiled coil peptides tested by ELISA using human sera, 43 were recognized by PNG (n = 42) sera at variable prevalence, however in all cases prevalence was >10%; 20 antigens displayed reactivity >29% (see Table S1). In addition, 17 peptides, which showed more than 30% of prevalence with PNG samples, were further tested with Colombian sera; all these peptides were antigenic with variable prevalence (Table 2). Ten peptides (*Pv*Pep27, *Pv*Pep42, *Pv*Pep43, *Pv*Pep45, *Pv*Pep*82.02*, *Pv*Pep82.03, *Pv*Pep83, *Pv*Pep95, *Pv*Pep96.01 and *Pv*Pep96.03) tested with the 90 human Colombian sera samples displayed a high degree of recognition, ranging from 30% to 86%. Recognition of the 17 peptides by PNG sera ranged between 29-71%, whereas recognition by Colombian sera for the same 17 peptides varied between 2–86%.

**Table 2:** Prevalence of antibodies reactive to *P. vivax* coiled coil fragments in volunteers from PNG and Colombia.

		PNG (n = 42)^a^			Colombia (n = 90)^a^				
		Seroprevalence*^b^*		Ratio>2	Seroprevalence		Ratio>2		
Peptide	Protein	n	Percentage	Percentage*^c^*	n	Percentage	Percentage	p value*^d^* seroprevalence	p value^d^ ratio>2
*Pv*Pep5	PVX_003585	15	36	24	8	9	5	0.0004	0.0016
*Pv*Pep27	PVX_113335	25	60	43	66	73	24	ns	0.0421
*Pv*Pep40	PVX_119385	13	31	17	8	9	1	0.0021	0.0014
*Pv*Pep42	PVX_087730	12	29	26	69	77	24	0.0001	ns
*Pv*Pep43	PVX_089660	23	55	43	27	30	13	0.0075	0.0002
*Pv*Pep45	PVX_123385	24	57	33	39	43	15	ns	0.0194
*Pv*Pep52	PVX_123480	18	43	19	25	27	16	ns	ns
*Pv*Pep63	PVX_118160	23	55	26	12	13	2	0.0001	0.0001
*Pv*Pep82.02	PVX_122740	15	36	24	40	44	15	ns	ns
*Pv*Pep82.03	PVX_091910	20	48	26	77	86	36	0.0001	ns
*Pv*Pep83	PVX_087730	22	52	33	48	53	17	ns	0.0421
*Pv*Pep90	PVX_000725	13	31	19	18	20	7	ns	ns
*Pv*Pep95	PVX_117455	17	40	29	39	43	11	ns	0.0220
*Pv*Pep96.01	PVX_124060	27	64	43	25	28	20	0.0001	0.0110
*Pv*Pep96.03	PVX_084385	30	71	60	36	40	12	0.0013	0.0001
*Pv*Pep101	PVX_085155	16	38	10	2	2	1	0.0001	0.0353
*Pv*Pep106	PVX_114430	17	40	36	25	28	9	ns	0.0004

a Human sera sample were tested at 1∶200 dilution. ^b^Corresponds to number and percentage of positive volunteers; Percentage of positive responses evaluated as OD values above the negative control mean + 3SD. Sera samples obtained from Swiss adult donors with no malaria history and no previous travel to malaria-endemic areas were used as negative control. *^c^*Percentage of OD ratio higher than 2 between the mean of the experimental and the mean of the control sera OD. *^d^*p value calculated by Fisher's exact test between PNG and Colombia. NS = not significant (p>0.05).

Interestingly, seven of the 17 selected peptides were the most antigenic (>50% of responders) in PNG (*Pv*Pep27, *Pv*Pep43, *Pv*Pep45, *Pv*Pep63, *Pv*Pep83, *Pv*Pep96.01, *Pv*Pep96.03), four peptides (*Pv*Pep27, *Pv*Pep42, *Pv*Pep82.03 and *Pv*Pep83) were the most reactive with Colombian sera (Table 2). Differences in reactivity were also observed between the two malaria-endemic sites in Colombia, Tierralta and Tumaco (data not shown). Responses against 16/17 peptides were stronger with PNG as compared with Colombian sera, presenting with OD ratios >2 (Table 2).

### Immunogenicity of α-helical coiled coil peptides in mice

**Figure 2:**
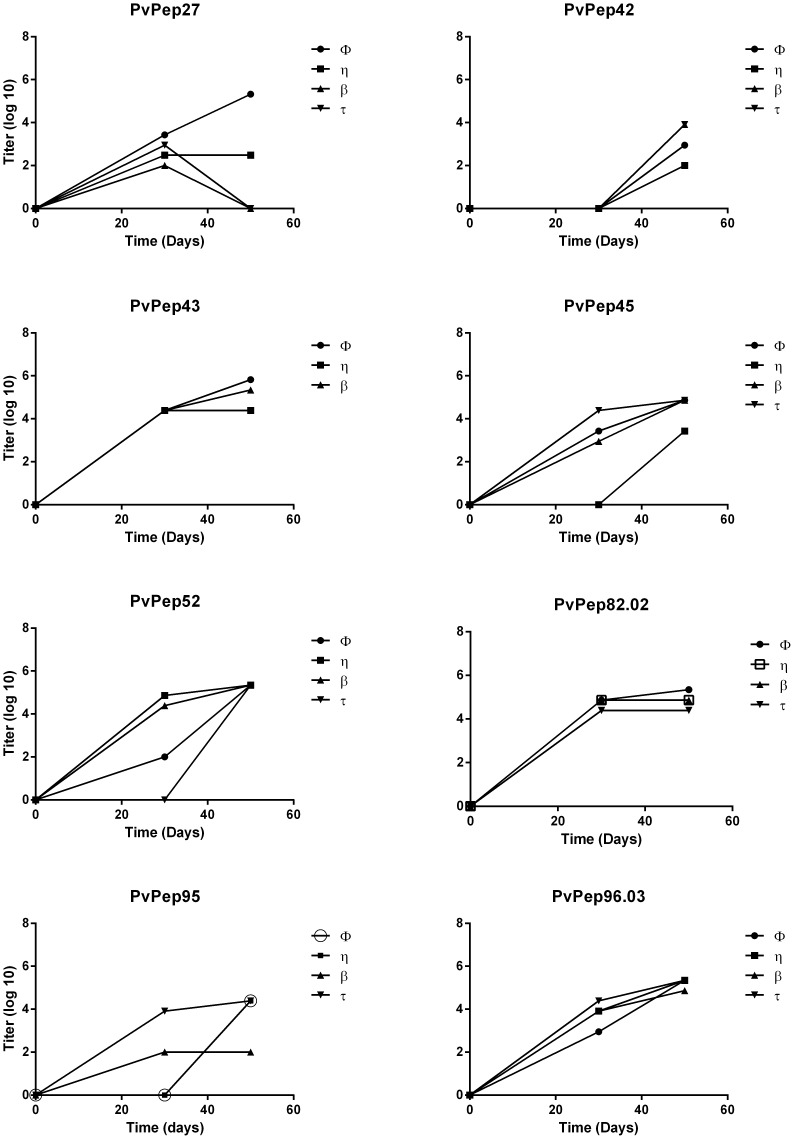
Immunogenicity of coiled coil peptides in BALB/c mice. Titration of IgG antibody responses to coiled coil peptides in immunized mice. Evaluation on days 0, 30 and 50. Titers shown are according to a Log_10_ scale. “φ, η, β and τ corresponds to an identification mark for each one of the animals per group. ELISA experiments were performed by duplicated in two independent experiments.

Eight peptides that showed prevalence >50% either with PNG or Colombian sera were further tested for their immunogenicity in BALB/c mice. Immunized mice developed specific IgG antibodies to the α-helical coiled coil fragments after the second immunization dose as determined by ELISA with the exception of those immunized with *Pv*Pep42; three immunization doses were needed to produce detectable antibody levels (Figure 2). Antibody titers increased steadily with titers ranging from 9×10^2^ to 2×10^6^ after the third immunization (Table 3). Mice immunized with *Pv*Pep27 and *Pv*Pep95 showed variable responses that were not uniform in all animals; two animals in each group failed to develop the typical boosting response after third dose. Antibody titers decreased and became negative (*Pv*Pep27) or remained stable (*Pv*Pep95); neither recognized the native protein in the IFAT (Table 3).

**Table 3:** Immunogenicity of *P. vivax* coiled coil fragments in BALB/c mice.

Antigen	Protein ID^a^	ELISA titer range	ELISA responders		IFA^b^
			n	Percentage	1∶20
*Pv*Pep27	PVX_113335	3.0×10^2^–2.4×10^4^	2	50%	−
*Pv*Pep42	PVX_087730	9×10^2^–8×10^3^	3	75%	−
*Pv*Pep43	PVX_089660	6.6×10^5^–2.0×10^6^	3	100%	+
*Pv*Pep45	PVX_123385	2.7×10^3^–2.2×10^5^	3	75%	++
*Pv*Pep52	PVX_123480	7.2×10^4^–2.0×10^6^	4	100%	−
*Pv*Pep82.02	PVX_122740	2.4×10^4^–2.2×10^5^	4	100%	++
*Pv*Pep95	PVX_117455	2.4×10^4^–7.2×10^4^	3	75%	−
*Pv*Pep96.03	PVX_084385	7.2×10^4^–2.2×10^5^	4	100%	+

a ID from PlasmoDB; ^b^(-) negative, (+) positive with 1-10, and (++) positive between 10 to 20 fluorescent parasites per well, respectively.

However, sera from four of the eight immunized groups were able to recognize native protein on *P. vivax* asexual blood stages in IFAT assays at a 1∶20 dilution; two showed strong reactivity (Table 3). Control mice, which received only adjuvant in saline solution, were non-responsive as indicated by ELISA and IFAT (data not shown).

### Cross-reactivity tests

**Figure 3:**
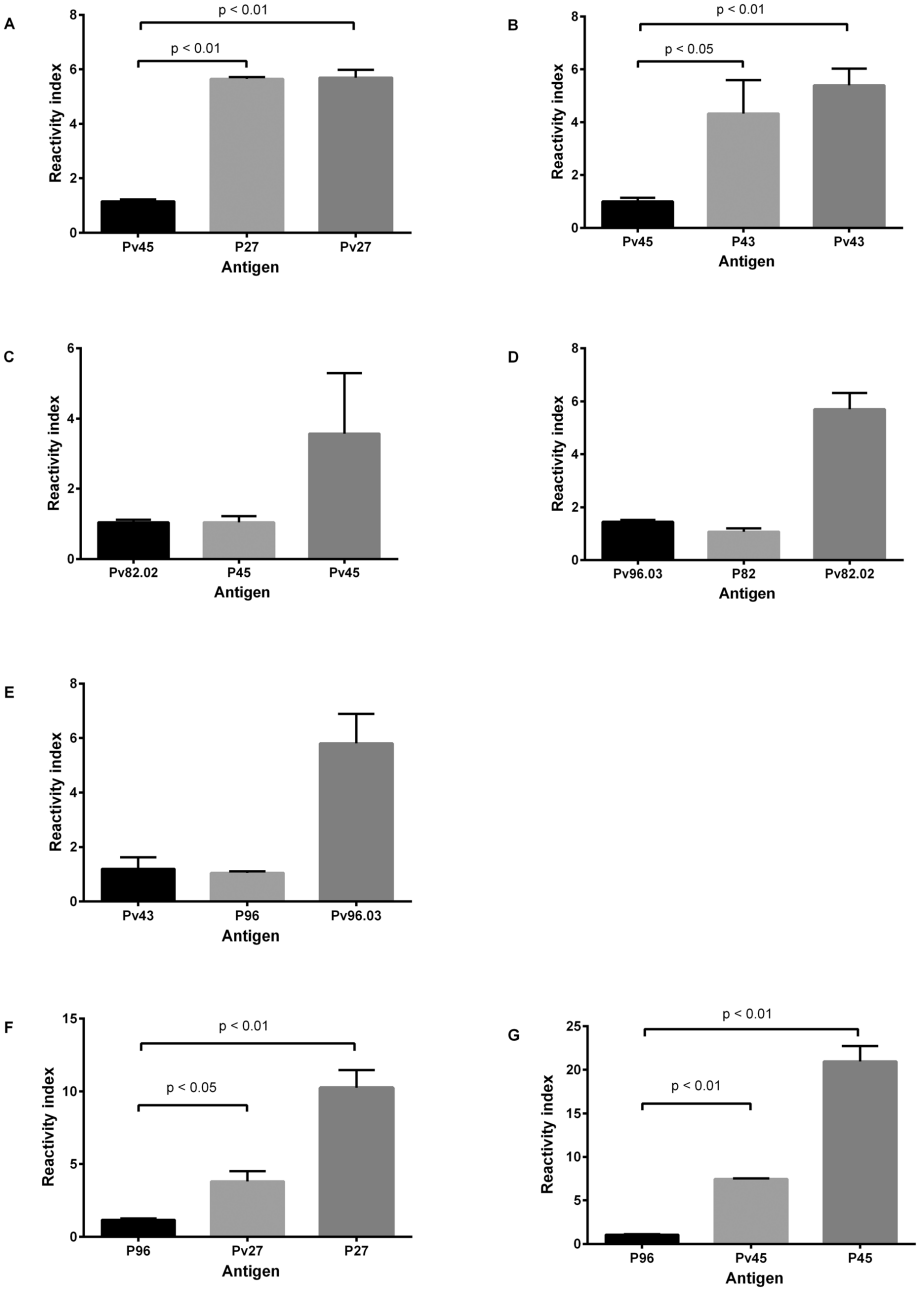
Cross-reactivity of *P. vivax* and *P. falciparum* antigens. Antigenic cross-reactivity between *P. vivax* and *P. falciparum* was tested by ELISA, testing mice sera samples **A**. anti-*Pv*Pep27; **B**. anti-*Pv*PepP43; **C**. anti-*Pv*Pep 45; **D**. anti-*Pv*Pep82 and **E**. anti-*Pv*Pep96; at 1∶200 dilution with the corresponding *P. vivax* and *P. falciparum* antigens. Additionally, affinity-purified human antibodies specific to **F**. *Pf*P27 and **G**. *Pf*P45 were used to test the reactivity of homologous *P. falciparum* and *P. vivax* antigens. Human IgG was tested at a 1∶200 dilution. In all cases, a non-related antigen was used as a negative control. Reactivity index defined as OD values of tested sample divided by the cut-off value, are reported as mean ± SEM for each mouse serum. Cross reactivity experiments were performed in duplicate in two independent experiments.

Sera from mice immunized with *Pv*Pep27 and *Pv*Pep43 were reactive with the corresponding orthologues *Pf-*P27 and *Pf-*P43 with similar reactivity indices as compared to a control sample (Figure 3). None of the other antigens (*Pf*-P45, *Pf*-P82 or *Pf*-P96) showed significant cross-reactivity. Moreover, cross-reactivity was also observed when specific affinity-purified human IgG to *Pf*-P27 and *Pf*-P45 were tested with the corresponding *P. vivax* orthologue; three-fold less reactivity was observed in both cases as compared with the positive control. Additionally, cross-reactivity with whole *P. falciparum* parasites was observed by IFAT (Table 4). No relationship was observed between homology and reactivity since fragments with low identity, such as *Pv*Pep45, were highly reactive with both the *P. vivax* fragment and the *P. falciparum* parasite, whereas *Pv*Pep82.02 with greater than 60% homology was not reactive (Table 4).

**Table 4:** Reactivity of IgG tested with different parasite antigen fragments and whole parasites.

Origin	Antibody	Identity^a^ (%)	*P. vivax* fragments^b^	*P. falciparum* fragments^c^	*P. vivax* parasite^d^	*P. falciparum* parasite^e^
Mouse	anti *Pv*Pep27	63	+	+	-	-
Mouse	anti-*Pv*Pep43	82	+	+	+	-
Mouse	anti-*Pv*Pep45	44	+	-	+	-
Mouse	anti-*Pv*Pep82	61	+	-	+	-
Mouse	anti-*Pv*Pep96	43	+	-	+	-
Human	anti *Pf*-P27	NA^d^	+	+	+	+
Human	anti *Pf*-P45	NA	+	+	+	+

a Identity between *P. vivax* and *P. falciparum* orthologous antigens; ^b^Reactivity tested by ELISA test using *P. vivax* antigens; ^c^Reactivity tested by ELISA test using *P. falciparum* antigens; ^d^Reactivity tested by IFA test with *P. vivax* blood stages; ^e^Reactivity tested by IFA test with *P. falciparum* blood stages; ^d^Does not apply.

### Cellular immune responses in mice

**Figure 4:**
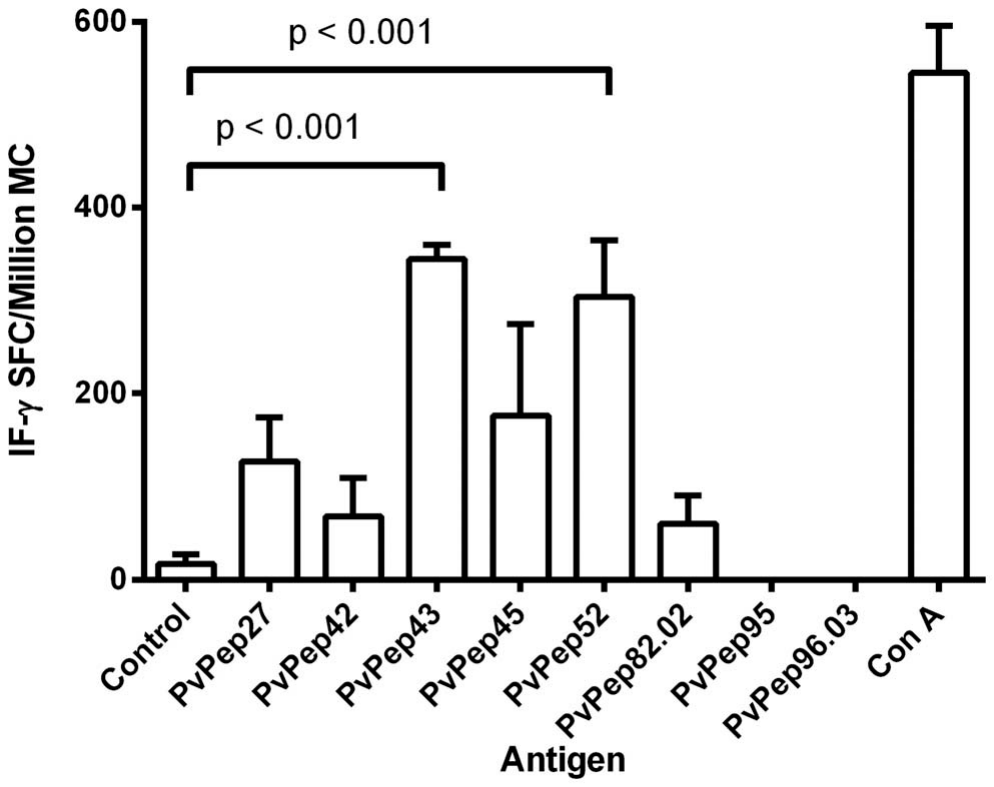
Production of IFN-γ by mononuclear cells from immunized mice. Proliferative responses of mononuclear cells obtained from mice immunized with eight synthetic peptides, and further *in vitro*-stimulated with 10 µg/mL of corresponding coiled coil peptides. Conconavalin A (Con A) mitogen was used as a positive control. RPMI 1640 medium was used as a negative control. Data of SFC were reported as mean ± SEM for each peptide. P value was calculated by the ANOVA test. MN: mononuclear cells.

T-cell IFN-γ production was induced by six (*Pv*Pep27, *Pv*Pep42, *Pv*Pep43, *Pv*Pep45, *Pv*Pep52 and *Pv*Pep82.02) of the eight peptides tested by ELIspot; *Pv*Pep95 and *Pv*Pep96.03 were not recognized by murine lymphocytes (Table 3). The greatest IFN-γ production was induced by *Pv*Pep43 and *Pv*Pep52 (mean SFC 344.7±15.33 and 304±60.8, respectively) followed by PvPep45, *Pv*Pep27 and *Pv*Pep42 (mean SFC 176.7±98.1, 127.3±47.6 and 68.3±47.6, respectively) (Figure 4).

Additionally, the selected peptides presented potential CD4+ epitopes in their amino acid sequences as confirmed by bioinformatics analysis (Table 5). No apparent relation was observed between the affinity of the predicted epitope and the IFN-γ results obtained, when mouse epitopes were described (Table 5). Higher affinity, defined as the lower percentile rank, were observed for *Pv*Pep27 and *Pv*Pep82.02 epitopes. When the alleles from human were tested, higher affinity was observed in all cases compared with mouse epitopes, although differences were observed in the main epitopes found. Same epitopes predicted for mouse alleles could be present in human alleles but with lower affinity.

**Table 5:** Cell immune response and association with HLA II epitopes prediction.

Antigen	IFN-γ production SFC^a^		Mouse			Human		
	Mean	range	Peptide	Allele	Percentile rank	Peptide	Allele	Percentile rank
*Pv*Pep27	164	125–228	KKQNAEKELSVLKKN	H2-Iad	9.8	VLKKNYDAMSEEIEE	HLA-DQA1*0401/DQB1*0402	1.02
						KKQNAEKELSVLKKN	HLA-DPA1*0201/DPB1*0501	16.09
*Pv*Pep42	133	104–169	PDYYKKITTKLQNNI	H2-Iad	20.72	INNITNDINILKSSI	HLA-DRB1*0301	0.31
						PDYYKKITTKLQNNI	HLA-DRB5*0101	1.4
*Pv*Pep43	344	314–360	VKKLREELNKVTNEY	H2-Iad	43.01	TNEYDDFKNKLELLY	HLA-DRB1*0801	2.37
						VKKLREELNKVTNEY	HLA-DPA1*0201/DPB1*0501	9.14
*Pv*Pep45	254	191–339	NEAKEEVIEKKEEMT	H2-Ied	48.85	INKNISTINDDVDHI	HLA-DRB3*0101	0.01
*Pv*Pep52	277	122–375	NINETKITHLRNKIE	H2-Ied	32.44	INEQININETKITHL	HLA-DRB1*0701	0.35
						NINETKITHLRNKIE	HLA-DRB1*0827	4.29
*Pv*Pep82.02	69	36–142	NLDQKILELQASFTC	H2-Iad	7.93	NEIKQVIKKIEGLEK	HLA-DRB5*0101	0.39
						NLDQKILELQASFTC	HLA-DRB1*0102	0.39
*Pv*Pep95	NR^b^	NR	LKDLNDKIRNYDSII	H2-Ied	47.22	EKGLKDLNDKIRNYD	HLA-DRB3*0101	0.26
						LKDLNDKIRNYDSII	HLA-DRB5*0101	17.04
*Pv*Pep96.03	NR	NR	NDDVDHINSNINNIN	H2-Iab	42.28	INKNISTINDDVDHI	HLA-DRB3*0101	0.01
						NDDVDHINSNINNIN	HLA-DRB1*0102	12.61

a SFC: spot-forming colonies×10^6^; ^b^No response observed.

## Discussion

**Figure 5:**
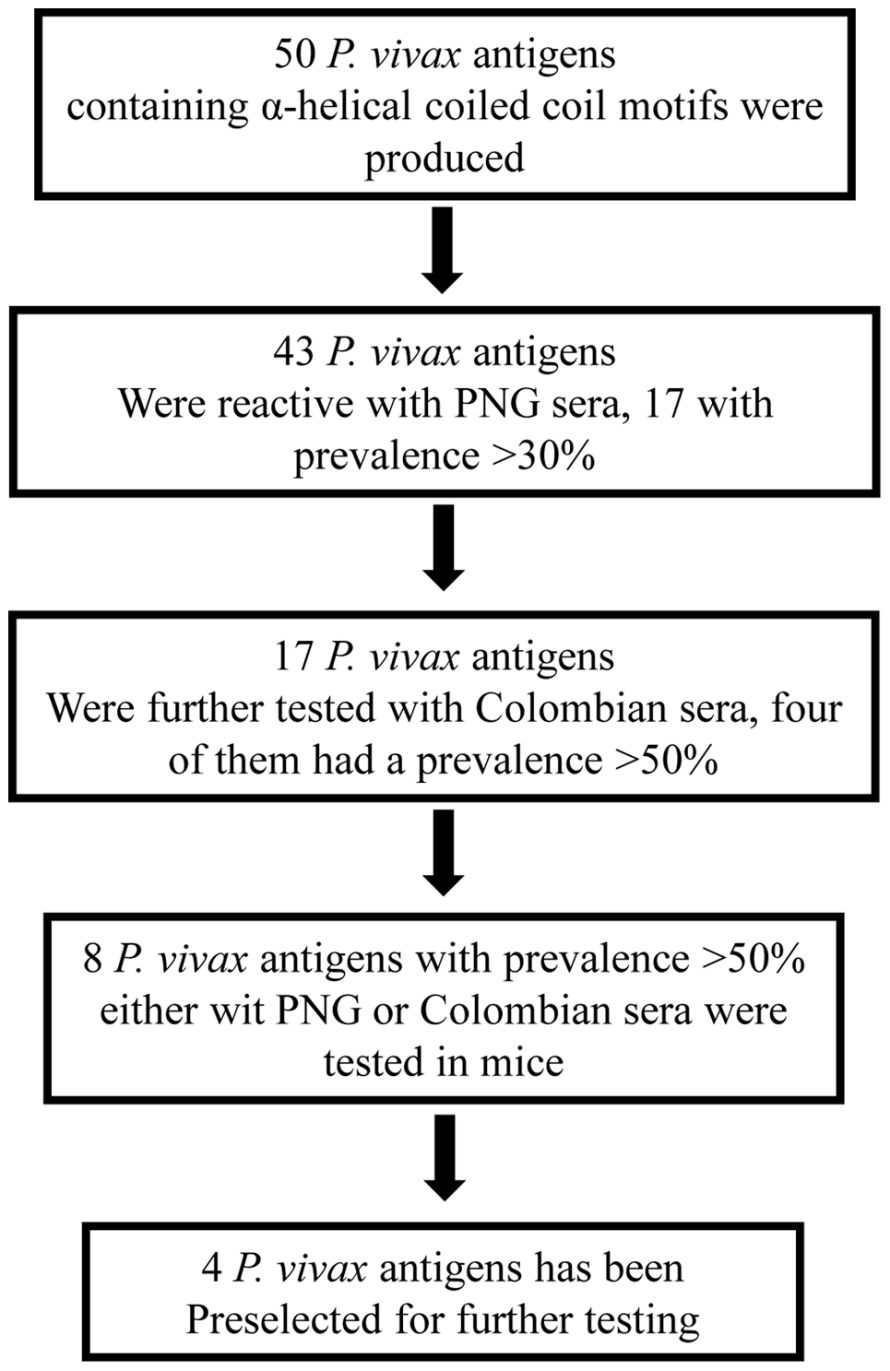
Schematic representation of the antigen selection process. Download selection is represented: first, 50 *P. vivax* antigens containing α-helical coiled coil motifs, selected from orthologues of *P. falciparum*, were chemically synthesized and tested with PNG sera. Seventeen reactive with prevalence >30% were tested with Colombian sera. Eight antigens with prevalence >50% either with PNG or Colombian sera were used for mice immunization and immune response testing. Four antigens were finally selected for further pre-clinical testing.

In an attempt to identify new target parasite antigens for malaria vaccine development, bioinformatics tools have been previously used to select proteins containing α-helical coiled coil motifs in *P. falciparum* proteins. In this study, similar algorithms were used in a pilot search of *P. falciparum* orthologous antigens in the *P. vivax* genome, and 50 α-helical coiled coil *P. vivax* segments showing a high degree of homology to the previously identified orthologous *P. falciparum* fragments were selected, and were further assess in antigenicity and immunogenicity studies; at the end four antigens were identified as potential targets for additional testing as vaccine candidates (Figure 5).

It is interesting to note that of the 50 fragments tested containing α-helical coils, 19 were recognized by sera of individuals living in *P. vivax* endemic areas of PNG and Colombia. Most of the fragments were antigenic with variable prevalence depending on the origin of the serum samples. Variation in reactivity among sera appeared to be associated mainly with the distinct malaria transmission conditions in these two regions [42], [43]. Whereas PNG is highly endemic for *P. vivax* and accounts for a large proportion of the malaria cases, Colombia is a low- to moderate malaria-endemic region where *P. vivax* is the prevalent *Plasmodium* parasite. However, other factors such as differences in the genetic background of the host and parasites, and transmission rate may also explain the differences observed in the recognition frequency. These results are similar to those found in previous studies where different reactivity was observed when antigens were tested with sera from different endemic areas [26], [44].

Additionally, it is very promising to find that eight peptide fragments were able to induce a significant antibody response in immunized mice with concomitant induction of IFN-γ producing T-cells with six of the peptides. Furthermore, specific antibodies to four of the fragments resulted in positive reactions in IFA assays using *P. vivax* blood- stage parasites; two of these antibodies were also reactive with *P. falciparum* orthologous antigens, although none was reactive to *P. falciparum* parasite antigens. On the other hand, affinity purified human antibodies specifics to two *P. falciparum* antigens were reactive with the *P. vivax* parasite antigens. All eight preselected antigens induced antibody responses although to a variable degree regarding antibody titers and antibody kinetics. Similar results were obtained in previous studies using *P. falciparum* orthologous antigens, which elicited variable intermediate-to- high antibody responses [26]. Responses do not seem to be associated with fragment length since strong antibody titers were observed in response to smaller fragments such as *Pv*Pep43. However, only four peptides (*Pv*Pep43, *Pv*Pep45, *Pv*Pep82.02, and *Pv*Pep96.03) induced antibodies in mice that were able to react with whole *P. vivax* parasites; these four peptides induced the strongest antibody responses. Peptides *Pv*Pep43 and *Pv*Pep82.02 are chromosome-associated proteins with the other two being hypothetical proteins.

Most interestingly, antibodies to *Pv*Pep43 were cross-reactive with the orthologous *P. falciparum* antigen, which could represent a clear advantage for multispecies malaria vaccine development provided that cross reactivity will be also observed with the *P. falciparum* parasite protein. Additionally, considering the interest on *Pf*-P27, previously described as a promising malaria vaccine candidate [26], we also tested the cross-reactivity to this antigen. Both sera from mice immunized with *Pv*Pep27 and specific purified human IgG were reactive with both *Pf-*P27 and *Pv*Pep27. Homology of the two peptides, *Pv*Pep27 and *Pv*Pep43, is variable (60% and 83%, respectively). Surprisingly, *Pv*82.02, which shares an identity of 60% with the corresponding orthologue, did not show cross-reactivity; interestingly, *Pv*Pep45 was shown to be reactive with purified human IgG anti-*Pf*-P45, however conversely, the *P. falciparum* antigen was not reactive with anti-*Pv*Pep45 mouse sera. None of the antibodies to *P. vivax* antigen tested showed cross-reactivity with the native protein in blood stages as detected by IFAT possibly due the lower sensitivity of the test due to a mixture of stages present in the donor's samples or the low protein expression.

Most of the peptides induced strong IFN-γ production as expected because of the presence of MHC-II epitopes predicted by bioinformatics analysis. *Pv*Pep43, *Pv*Pep45 and *Pv*Pep52 induced higher levels of IFN-γ along with strong antibody responses. *Pv*Pep95 and *Pv*Pep96.03 did not induce detectable IFN-γ production in agreement with the low affinity CD4+ cell epitopes predicted as assessed by the IEDB analysis resource Consensus tool. It is worthy to note that peptides inducing the greatest IFN-γ production also induced the strongest antibody responses, which indicates a great potential for vaccine development. Since not association was observed between mouse and human predicted epitopes, additional experiments should be performed in non-human primates to assess the cell immune response.

Most of the antigens that have trans-membrane segments or are involved in secretory pathways were found to be poorly antigenic, suggesting that these fragments may not be expressed on the parasite surface or are not present in sufficient concentrations to allow recognition. Further investigations are warranted to determine the actual localization of the corresponding antigens. Although most antigenic fragments were not associated with trans-membrane domains with only two (*Pv*Pep52 and *Pv*Pep96.01) involved in secretory pathways, it has been shown that soluble proteins released at the time of schizont rupture are equally effective at triggering immune responses [45]–[47].

Though desirable, the functional activity of antibodies elicited in mice or humans as measured by a parasite growth inhibition assay could not be performed due to the lack of *P. vivax in vitro* cultures. Further preclinical studies, including experimental infection in non-human primates, must be carried out to address this question. Taken together, present data, along with that previously published, point to coiled coil peptides as an important potential source of malaria vaccine candidates. Analysis of α-helical coiled coil motifs should be extended to the entire group of erythrocytic parasite antigens. Poly-subunit antigens should be designed, containing both relevant *P. vivax* and *P. falciparum* fragments that are capable of inducing effective immune responses. Thus, this study has direct relevance to *P. vivax* asexual blood- stage vaccine design and suggests that some of the antigens tested could be effective in different malaria settings such as PNG and Colombia.

## Supporting Information

Table S1 **Bioinformatics analysis of coiled coil fragments and Antibody response to PNG sera samples of all coiled coil** ***P. vivax*** **tested antigens.**
(XLSX)
